# Objective assessment of patellar maltracking with 3 T dynamic magnetic resonance imaging: feasibility of a robust and reliable measuring technique

**DOI:** 10.1038/s41598-020-72332-9

**Published:** 2020-10-08

**Authors:** Jannik Frings, Tobias Dust, Matthias Krause, Malte Ohlmeier, Karl-Heinz Frosch, Gerhard Adam, Malte Warncke, Kai-Jonathan Maas, Frank Oliver Henes

**Affiliations:** 1 Department of Trauma and Orthopaedic Surgery, University Medical Center Hamburg-Eppendorf, Hamburg, Germany; 2 Department of Diagnostic and Interventional Radiology and Nuclear Medicine, University Medical Center Hamburg-Eppendorf, Hamburg, Germany

**Keywords:** Experimental models of disease, Risk factors, Trauma, Musculoskeletal system, Magnetic resonance imaging, Bone imaging

## Abstract

Patellofemoral maltracking predisposes the patellofemoral joint to instability. The purpose of this study was to provide a reliable dynamic magnetic resonance imaging (MRI) measuring technique for patellofemoral maltracking, and to investigate the influence of anatomical risk factors (AF) on patellar maltracking. Ten patients (2 males,8 females, average 19 years) with clinical maltracking and 20 controls (10 males,10 females,average 28 years) were examined with a dynamic, multi-slice gradient-echo MRI sequence, during repetitive flexion(40°) and full extension, in an open-chain-active-movement. In a 30-s time frame three simultaneous transverse slices were acquired. Dynamic mediolateral translation and dynamic tilt were measured at defined positions, by two independent examiners. Reproducibility was tested in a set of five knees. Common AFs for patellar maltracking (tibial-tuberosity-to-trochlear-groove-(TT-TG)-distances, trochlea-sulcus-angle, trochlea-sulcus-depth, lateral-trochlear-inclination and Caton-Deschamps-Index) were analyzed in consensus, using standard static MRI sequences. In patients, dynamic mediolateral translation was significantly greater in patients (12.4 ± 6.9 mm vs. − 0.1 ± 2.3 mm, *p* < 0.001) and the patella was positioned significantly more laterally (17.5 ± 6.9 mm vs. 3.1 ± 2.4 mm, *p* < 0.001) compared to controls. During movement, the patella tilted 16.3 ± 13.1° laterally in patients and 1.9 ± 4.3° medially in controls (dynamic tilt) (*p* < 0.002). All AFs were significantly different between patients and controls. Pathological TT-TG-distances, Caton-Deschamps-Indices and trochlea-sulcus-angles strongly correlated with dynamic patellar translation and dynamic patellar tilt (*p* < 0.001). In the patient population, the primary pathologies for maltracking were lateralized-tibial-tubercle (n = 5), trochlea dysplasia n = 2, patella alta (n = 3). Interrater-reliability for translation and tilt-measurement was excellent (0.971/0.976, 95% CIs 0.939–0.986/0.950–0.988). Dynamic MRI reliably differentiates between abnormal and physiological patellar tracking. Dynamic tracking and tilt strongly correlate with measurable AFs, which reinforces their clinical use and validates the presented technique.

## Introduction

Several anatomical risk factors predispose the patellofemoral joint (PFJ) to instability by influencing its physiological kinematics^1–8^. The gold standard diagnostic tool for patellofemoral disorders is magnetic resonance imaging (MRI), as it provides quantifiable information about the distances between the tibial tuberosity and the trochlear groove (TT-TG)^9^ or the posterior cruciate ligament (TT-PCL)^10^, the patella height^11–14^ and the morphological properties of the trochlea^15,16^. Despite their predictive value^17^, the validity of these parameters depends on the image plane levels and orientation, as well as positioning of the knee inside the MRI coil^10,18^. Furthermore, conventional MRI images depict a static condition without giving information about patellofemoral kinematics. Dynamic evaluation of the PFJ is therefore, to this day, obtainable only by clinical examination, and its accuracy depends on the examiner’s experience^19^.

In order to overcome these limitations, previous studies have proposed kinematic MRI protocols, which comprise sequential static images of the PFJ, recorded in different flexion angles of the knee^20–23^. Effectively, these protocols allow no real dynamic evaluation and, most importantly, they lack consideration of the quadriceps muscle, which influences patellofemoral kinematics and stabilization^24,25^. The introduction of dynamic MRI protocols has provided a base for real-time imaging of patellar tracking^26,27^. However, the resulting measurements have some limitations^26–28^. One is that the necessary acquisition times are inconveniently long, another is the patient set-up hardly allows natural leg movement, since the utilized MRI sequencing techniques are prone to movement artefacts. Therefore, slow and controlled movement is often reinforced by extension against continuous resistance, which results in an unphysiological alteration of the contractile activity of the quadriceps^27^.

Furthermore, as the patella moves during knee flexion and extension, the effective field of view (FOV) is limited to few transverse slices in which the patella is completely visible. Consequently, visualization of the entire course of the patella requires retrospective repositioning. Consequently, dynamic mediolateral patellar translation is measured only at the lateral edge of the patella and cannot be referenced to its size and/or tilt^8,21^. The purpose of the present study was therefore, to provide a more convenient and reproducible measuring technique for dynamic patellofemoral imaging. We hypothesize, that anatomically referenced measurement of patella tracking, during natural, unobstructed knee movement allows reliable differentiation between physiological and pathologically altered patella tracking. Further, we aimed to investigate the influence of a larger number of relevant anatomical risk factors on PFJ tracking.

## Methods

### Subjects

All patients who presented with symptoms of patellofemoral instability and maltracking between December 2019 and January 2020 were examined and identified by an orthopaedic surgeon (J.F., M.K. or K.F.). Patients with the clinical suspicion of a PFJ maltracking were selected for subsequent dynamic MRI examination.

Inclusion criteria were a positive history of patellofemoral symptoms, such as recurring patella dislocations and/or a persisting feeling of subjective instability after a dislocation, a positive (reversed) J-sign indicating patellar engagement into the trochlea, an increased mediolateral translation and the provision of patients’ consent. Patients presenting with isolated patellar instability without signs of a maltracking, or patients with acute dislocations, concomitant disorders, or a limited range of motion (ROM) < 0°–90°, were not included.

Participants in the control group were selected from healthy volunteers without any knee-related health issues, previous knee surgeries or further impairment of the investigated extremity, who agreed to take part in the study. In addition, a prior clinical examination was mandatory, in order to exclude clinical signs of a patellar instability and/or maltracking, such as the J-sign or a clinically increased mediolateral patella translation.

### MRI technique and examination setup

All subjects were examined using a 3 T MRI unit (Ingenia, Philips, Best, Netherlands).

**Figure 1:**
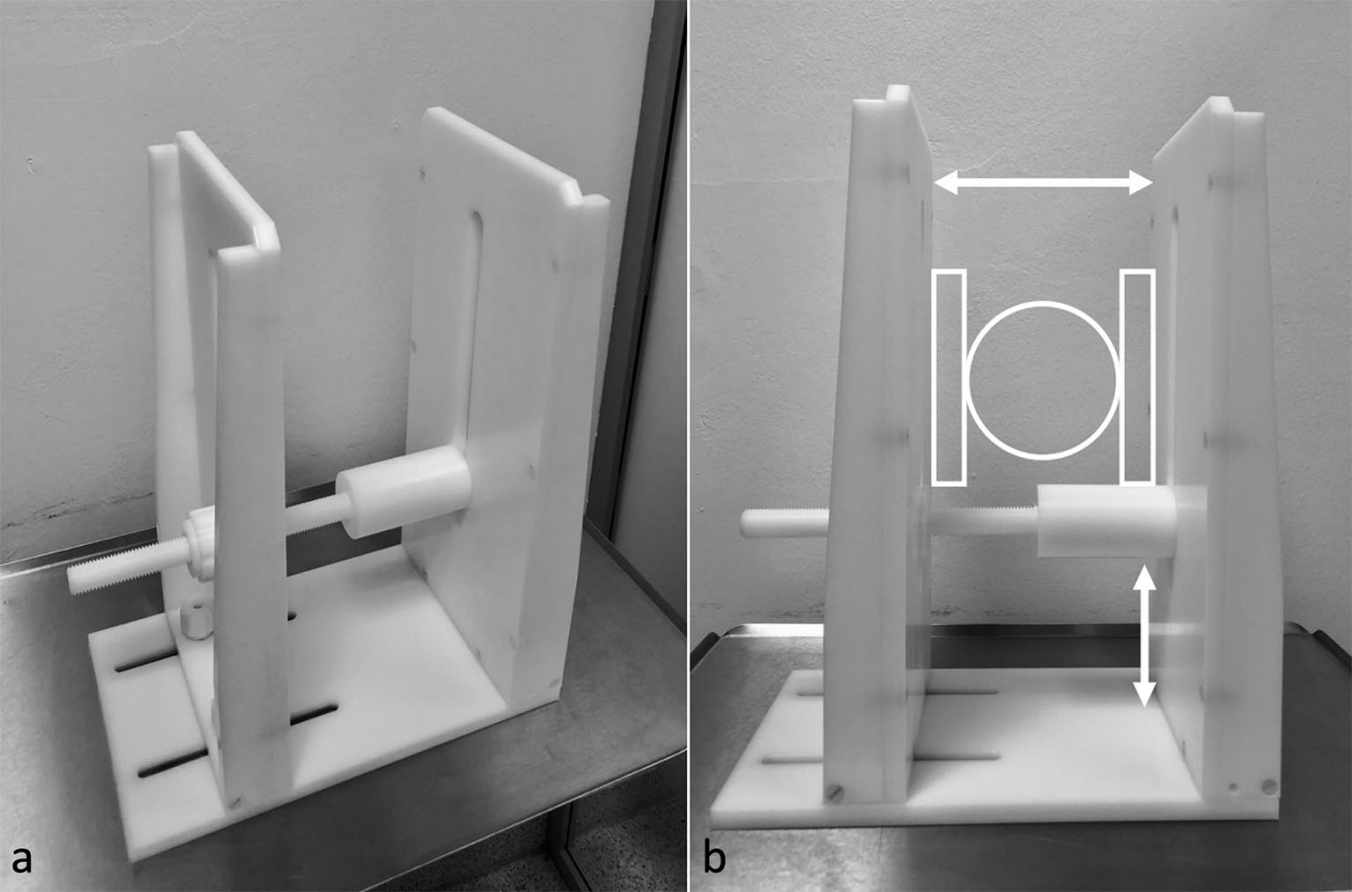
Custom-built and MRI-safe patient positioning device. A custom-built and MRI-safe patient positioning device was used to secure the knees in variable flexion angles. The fully adjustable design enabled a flexible utilization, regardless of the size and thickness of the knee. The knee (circle) is positioned in the middle of the positioning device, while the ring coils are placed on both sides of the knee (rectangles).

**Figure 2:**
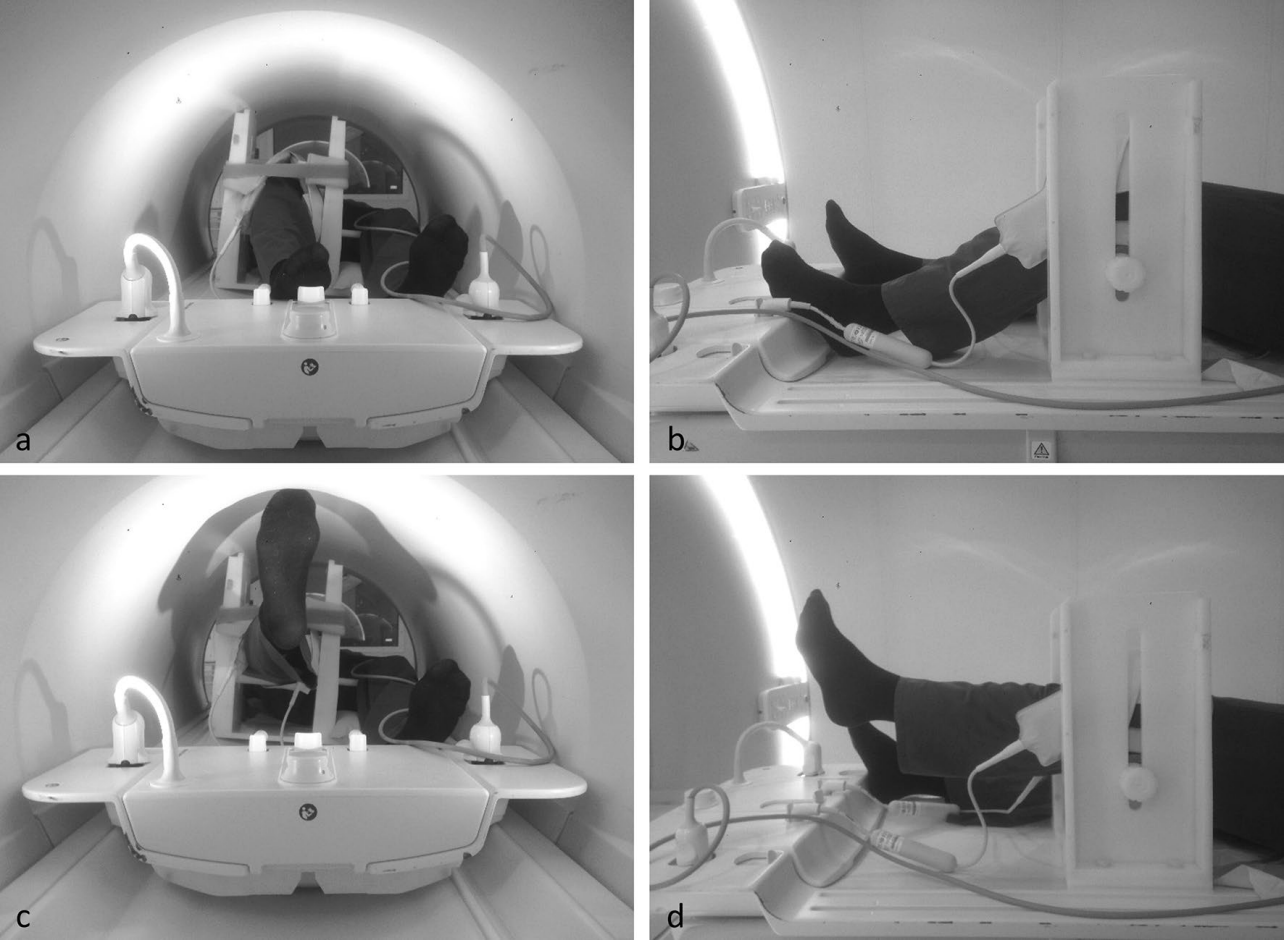
Patient positioning in the custom-built positioning device and range of motion. With patient in supine position the knee was placed inside the adjustable positioning device, braced by the ring coils. The femur was strapped to the device to prevent excessive movement during acquisition. With this setup a ROM between 30° and 40° of flexion (**a** + **b**) and full extension (**c** + **d**) was achievable.

Participants were placed in a supine position and a standard protocol was performed that included a fat-saturated PDw Fast Spin Echo sequence (FSE) in frontal, sagittal, and transverse plane orientations, as well as a T1w FSE sequence in coronal orientation was performed, using a 16-channal knee coil (Philips, Best, Netherlands). The acquisition time for the static images was 13 min and 17 s. The dynamic examination protocol was performed using two ring coils, which were fixed in a customized and individually adjustable dynamic examination device (Fig. 1). In the resting position, the knee was safely stored in 40° of knee flexion. The participants were then asked to perform an active, open-chain motion sequence, slowly extending and flexing their knees twice consecutively, in a pre-set range of motion from 40° to 0° of knee flexion, during real-time acquisition of the cinematic sequences (Fig. 2). Unlike movement against resistance, as described in previous studies^27,29^, a non-obstructed open-chain motion sequence was preferred, to allow a natural, non-artificial patellar tracking. The time frame for active flexion and extension of the knee was 30 s.

For dynamic imaging, a multi-slice spoiled gradient echo sequence with three slices in transverse orientation was used, which were followed by one sequence in sagittal plane orientation^30,31^, with a SENSE-factor of 2, a repetition time (TR) set to 8.3 ms and an echo time (TE) set to 1.32 ms. A pulse angle of 8° was chosen for optimal contrast. Slice thickness was set to 7 mm, with an acquired in-plane voxel size of 1.6 mm × 1.6 mm, reconstructed to 0.83 mm × 0.83 mm. Temporal resolution was set to 0.6 s, so a total of 150 images was acquired during the transversely oriented dynamic sequence, with a total scanning time of 30 s, to allow smooth and continuous limb movement during acquisition. The full range of patellar tracking was assessed by acquiring three transverse slices simultaneously. They were located at the levels of the posterior femur condyles, the deepest point of the trochlear sulcus, and the largest diameter of the patella in maximum extension. Thereby, gap size of 12 mm was found to be most sufficient, to allow full coverage of all relevant anatomic landmarks, while tracking the kinematics of the patellofemoral joint.

The total acquisition time for the dynamic MRI examination was one minute and 35 s.

### MRI analysis and measurement

MRI analysis was performed with Centricity RIS/PACS (GE, Boston, USA).

Radiological parameters of anatomical risk factors were assessed on static transverse and sagittal MRI images. Patella height was determined using the Caton-Deschamps Index (CDI) on sagittal MRI images, with the relaxed knee placed in 20° of flexion, using a standard MRI knee coil^14,32^.

The distance between the tibial tubercle and the trochlear groove (TT-TG) was measured in accordance with Schoettle et al.^9^, with the knee placed in the same position. Due to its dependence on joint positioning (tibial rotation), the distance between tibial tubercle and medial boarder of the posterior cruciate ligament (TT-PCL) according to Seitlinger et al.^10^ was additionally assessed.

The bony trochlear morphology was assessed on the first proximal transverse MRI plane, which displayed a fully cartilage covered trochlea^33,34^. Accordingly, three different parameters were measured: the trochlear sulcus angle, lateral trochlear inclination (LTI), and trochlear depth. The sulcus angle was defined by the angle between the lateral and the medial facet of the trochlea. The lateral trochlear inclination was measured as the angle between the femoral posterior condylar line and the lateral trochlear facet^16^. The distances between the posterior condylar line and the medial (MCD) and lateral condyles (LCD), as well as the lowest part of the trochlear groove (LTG) were measured. Trochlear depth was defined to be: (MCD + LCD)/2—LTG.

**Figure 3:**
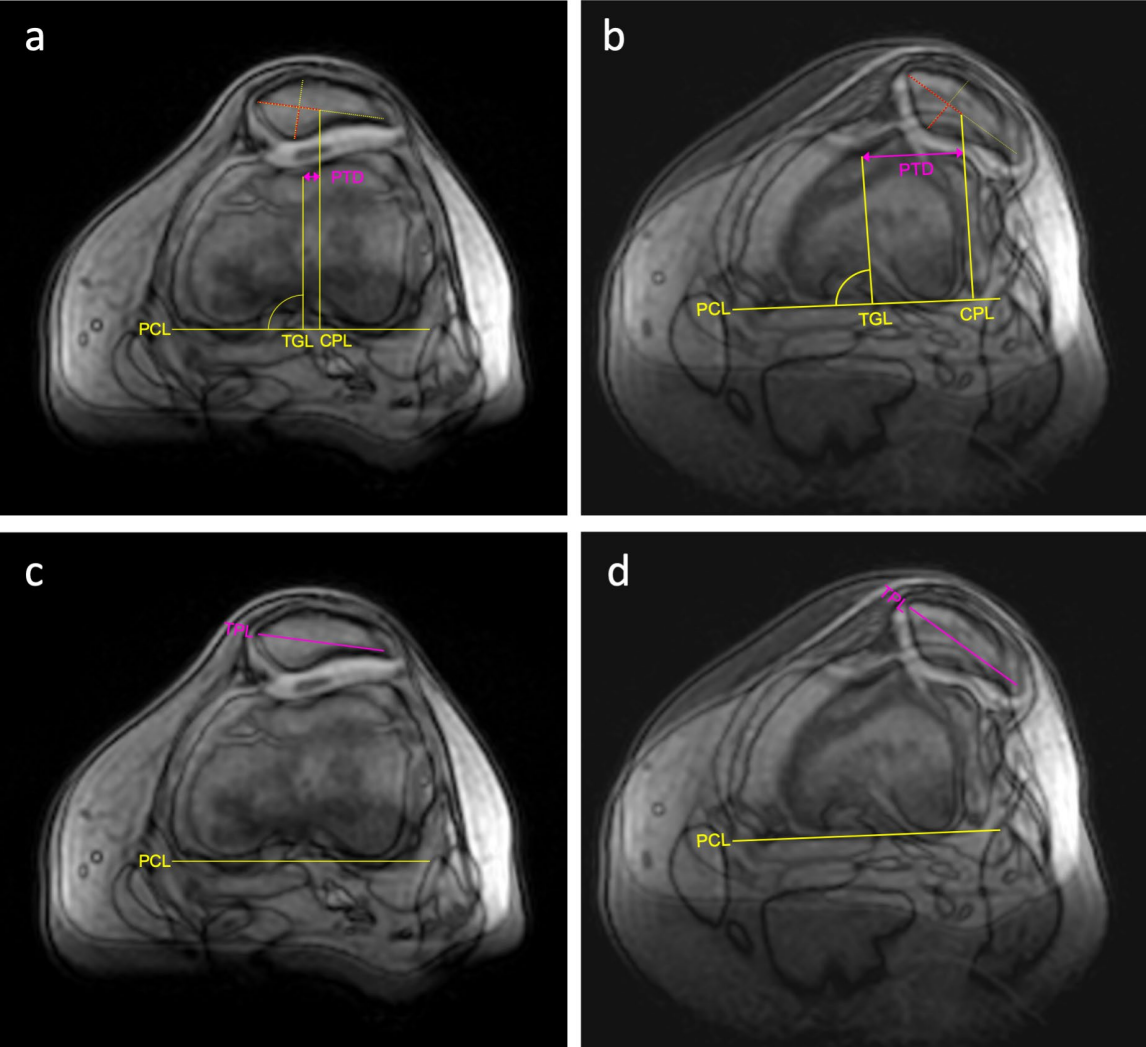
Dynamic measurement of patellar tracking and tilt in a female patient with patella maltracking. For measurement of dynamic mediolateral patellar translation, corresponding slices in transverse orientation of the same knee position were selected to provide clear visualization of all relevant reference landmarks, such as the posterior condylar line (PCL) and the trochlear groove, and to generate a complete picture of the patella. For presentation purposes the matched slices were superposed in this figure. Patellar tracking was measured in relation to the posterior condylar line (PCL). The patellotrochlear distance (PTD) was measured between a perpendicular line to the trochlear groove (TGL) and another to the center-center position of the patella (CPL) (**a** + **b**). Patellar tracking was calculated from the difference between the PTD in 30° of flexion (**a**) and in extension (**b**). The angle between the PCL and a transpatellar line was defined to be the patellar tilt (**c** + **d**). The dynamic patellar tilt was calculated from the difference between the angle in 30° of flexion (**c**) and in extension (**d**).

Dynamic mediolateral translation of the patella was measured by implementing a novel measuring technique designed relative to the size of the patella. First, the maximum patella width was measured at defined points of the medial and lateral border and the center–center position of the patella was marked. With the knee in flexion, a line perpendicular to the posterior condylar line was drawn through the deepest point of the sulcus and another through the center–center of the patella. The distance between the two lines was measured, to determine the patella position in flexion (Fig. 3a).

The entire procedure was then repeated using a transverse image with the knee in full extension, and the distance between the center–center of the patella and the current position of the deepest point of the sulcus was measured, after re-creating the posterior condylar line (Fig. 3b). Thus, the measurements were free of influence from possible movements of the femur during extension and flexion.

The difference between the patellotrochlear distance in extension and in flexion was then used to calculate dynamic mediolateral translation. The patellar tilt was measured accordingly, by determining the angle between individual posterior condylar lines and the mediolateral diameter of the patella, crossing the center–center position (Fig. 3c,d).

To test the reproducibility of the measurement technique a set of five randomly selected knees was appointed for a second independent dynamic knee MRI. The two different measurements of dynamic mediolateral translation and dynamic tilt were compared and the reproducibility was calculated, respectively.

All measurements were made by a musculoskeletal radiologist with six years of experience (J.M.) and by an orthopaedic surgeon with six years of experience in radiological measurement of the patellofemoral joint (J.F.) to verify interrater agreement of this measuring technique.

### Statistical analysis

Statistical analysis was performed with Microsoft Excel, version 16.31 (Microsoft, Redmont, WA, USA) SPSS Statistics, version 21.0 (IBM, NY, USA) and a descriptive analysis was conducted. Normal distribution was verified using the Kolmogorov–Smirnov test. Interrater reliability and reproducibility or the measurement technique were tested by calculation of the intraclass correlation coefficients (ICC)^35^. Differences between the mean values for dynamic mediolateral translation and dynamic patellar tilt were calculated using the paired t-test to calculate p values. Pearson correlation was calculated between radiological parameters and dynamic mediolateral tracking as well as dynamic patellar tilt. Correlation plots were built with Microsoft Excel. Since the presented study reports on the feasibility of a novel technique, no prior power analysis was performed.

### Ethical statement

The study protocol was approved by the by the Ethics Committee of the Medical Chamber in Hamburg, Germany (ID PV7101) and conducted according to Good Clinical Practice guidelines and the recommendations of the Declaration of Helsinki. Written informed consent was obtained from each patient (or parents, if underaged) and participants who decided to participate before sample acquirement.

## Results

Ten patients (2 males vs. 8 females, mean age 19 ± 10 years) with a symptomatic patella maltracking and 20 asymptomatic healthy controls (10 males, 10 females, mean age 28 ± 6 years) were enrolled in the study. The primary pathologies of included patients were lateralized tibial tubercle (n = 5), trochlear dysplasia n = 2 and patella alta (n = 3). All radiological parameters of anatomical risk factors among the patients showed significant differences to those of the control group (Table 1).

**Table 1:** Mean data of subjects and quantitative MRI measurements of the PFJ.

Parameter	Control (n=20)	Case (n=10)	*p*
**Side**			
Right knees	13	3	
Left knees	7	7	
**Gender **			
Male	10	2	
Female	10	8	
**Age **(min–max) Years	28.2 (23–50)	19.4 (12–36)	
**TT-TG **(± SD) mm	9.9 ± 3.3	18.8 ± 5.2	< 0.001
**TT-PCL **(± SD) mm	18.2 ± 3.1	21.7 ± 3.9	0.025
**Trochlea sulcus angle **(± SD) Degrees	131.5 ± 6.4	148.6 ± 14.4	0.004
**Trochlea sulcus depth **(± SD) mm	7.2 ± 1.6	4.9 ± 1.9	0.006
**Lateral inclination angle **(± SD) Degrees	26.1 ± 3.9	14.7 ± 7.3	0.001
**Caton Deschamps Index **(± SD)	1.1 ± 0.1	1.4 ± 0.2	0.003
Patellar position in flexion) (± SD) mm	3.2 ± 1.9	5.1 ± 4.2	0.192
Patellar position in extension (± SD) mm	3.1 ± 2.4	17.5 ± 6.9	< 0.001
Dynamic mediolateral translation (± SD) mm	−0.1 ± 2.3	12.4 ± 6.9	< 0.001
Patellar tilt in flexion (± SD) Degrees	7.9 ± 3.4	7.6 ± 6.5	0.894
Patellar tilt in extension (± SD) Degrees	6.1 ± 4.6	23.9 ± 12.5	0.001
Dynamic patellar tilt (± SD) Degrees	1.9° ± 4.3° to medial	16.3° ± 13.1° to lateral	0.002

*TT-TG* tibial tuberosity-trochlear grove distance, *TT-PCL* tibial tuberosity-posterior cruciate ligament distance, *CDI* Caton-Deschamps Index, *SD* standard deviation.

**Figure 4:**
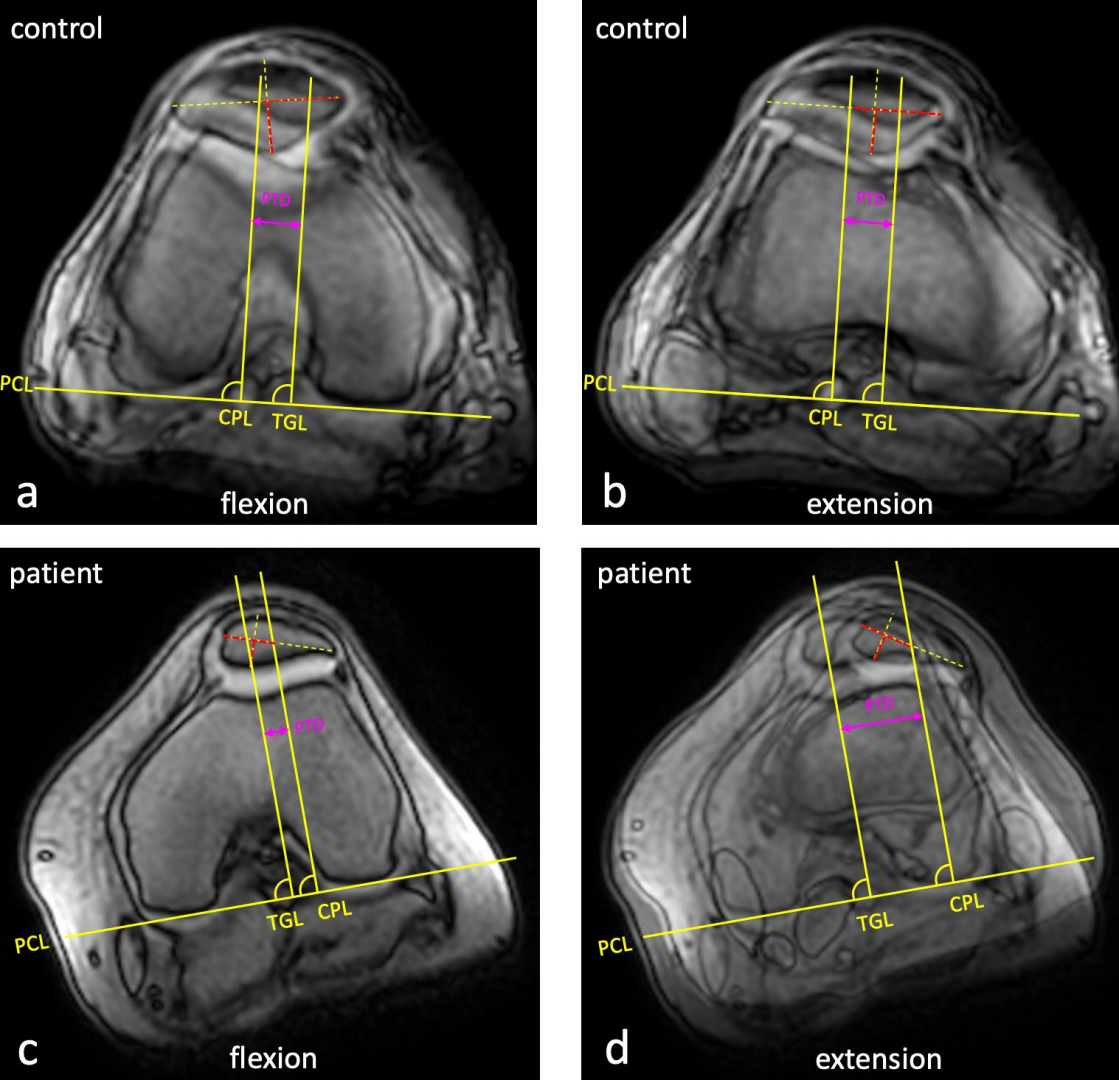
Measurement of dynamic mediolateral patellar translation in healthy controls (**a** + **b**) and symptomatic patients (**c** + **d**). PCL: posterior condylar line, CPL: perpendicular line to the center-center position of the patella, TGL: perpendicular line to the trochlear groove, PTD: patellotrochlear distance.

**Figure 5:**
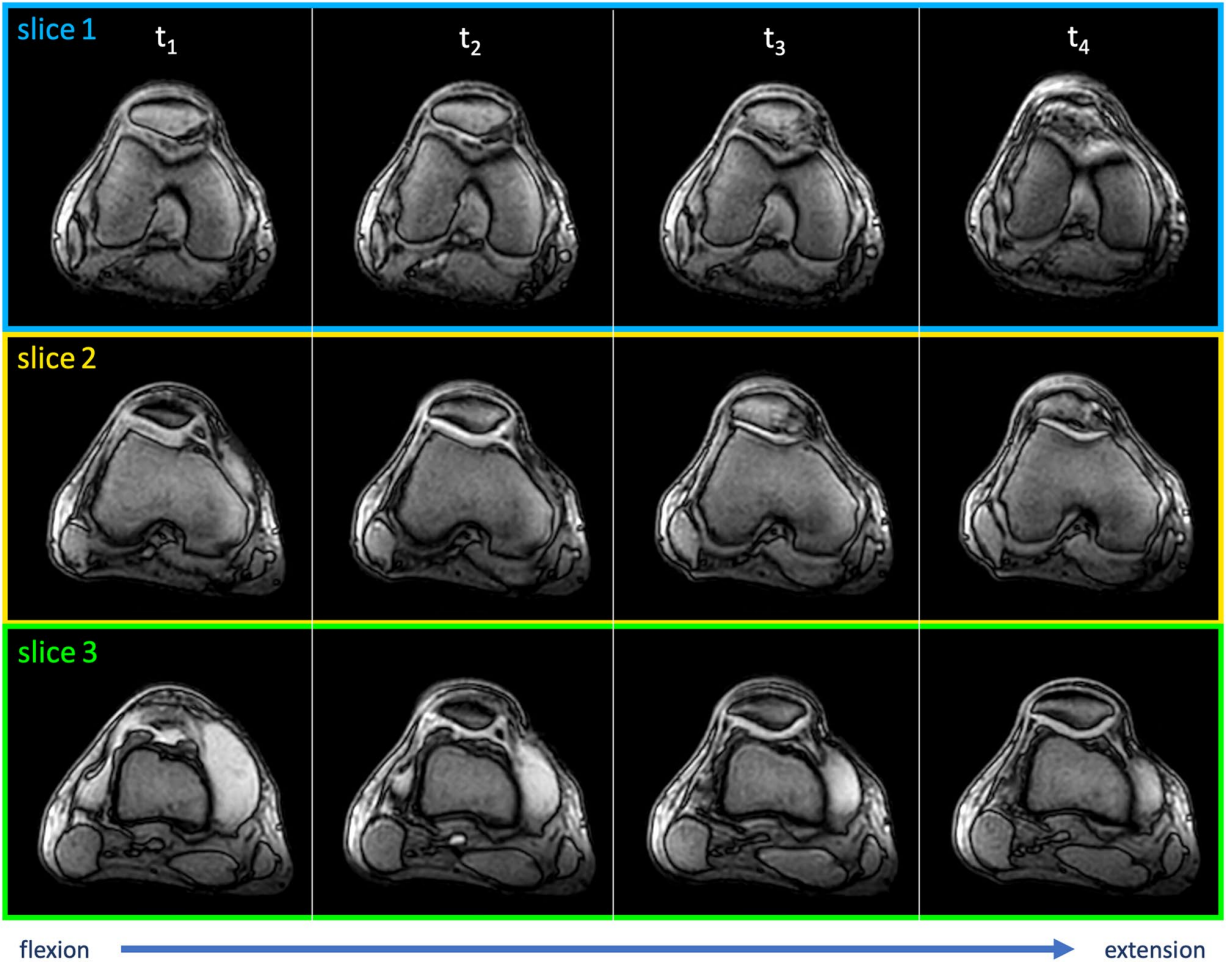
Physiological dynamic mediolateral patellar translation in a right control knee. During active movement of the knee from flexion to extension, three parallel transversely oriented slices were simultaneously scanned to allow continuous visualization of mediolateral patellar tracking. Therefore, the patellar position was measured in several corresponding points in time (t). Slice 1 was located at the level of the posterior femur condyles, slice 2 at the level of the deepest point of the trochlear sulcus, and slice 3 at the level of the largest diameter of the patella in maximum extension. Gap size was set to 12 mm.

**Figure 6:**
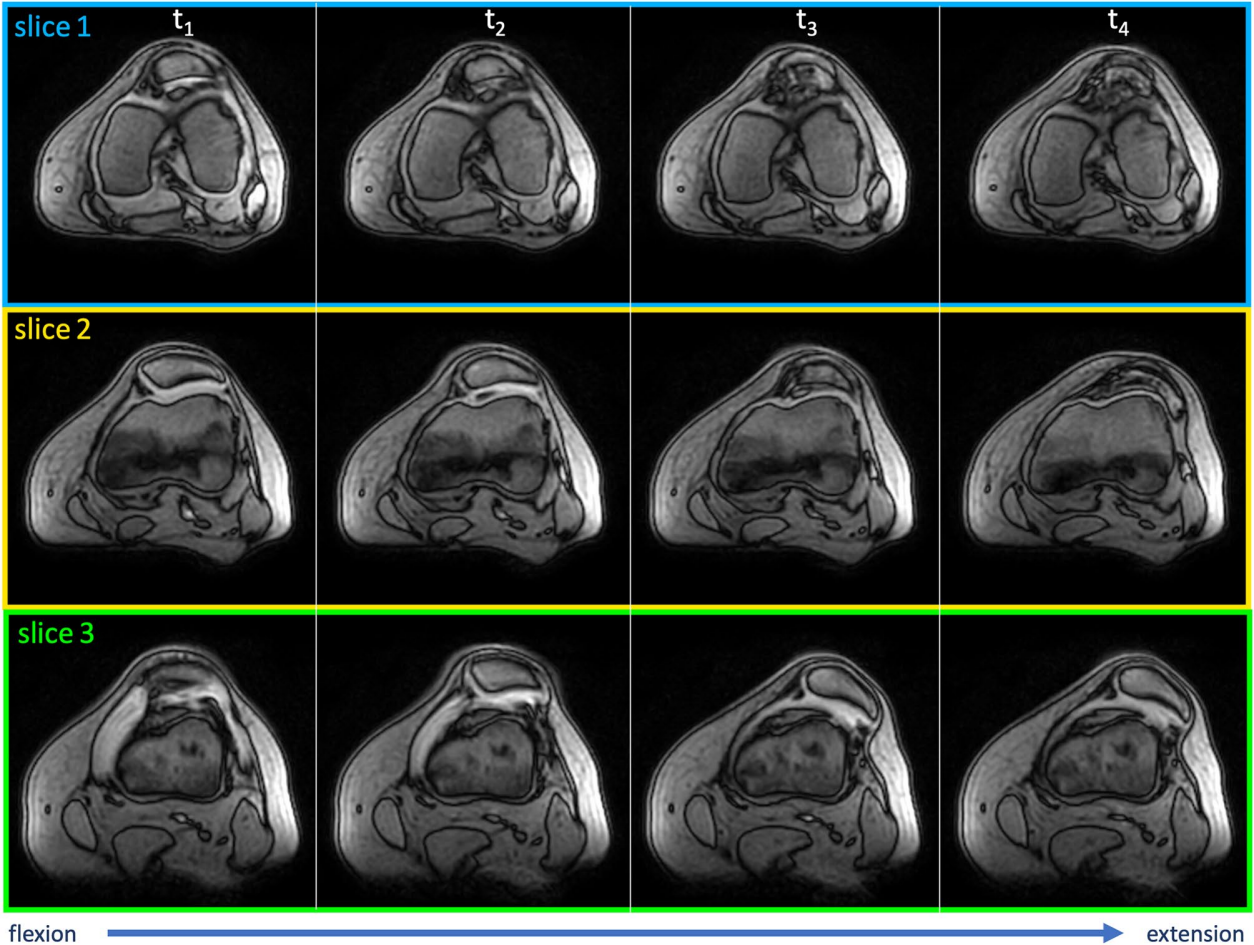
Patellar maltracking in a symptomatic left patient knee. Compared to the controls, the dynamic mediolateral patellar translation was significantly greater in the patient group. Furthermore, with continuous extension of the knee, the patella tilted significantly more laterally.

In extension, the patella was located significantly more laterally (*p* < 0.001) and the distance of dynamic mediolateral translation was significantly greater in the symptomatic group than in the controls (*p* = 0.001) (Fig. 4) Furthermore, the patella was tilted significantly more laterally in extension (*p* = 0.001), thereby showing a significantly larger dynamic range in the symptomatic group (*p* = 0.002) (Table 1). No significant differences were noted between the groups for the patella positioning (*p* = 0.192) or patella tilt in flexion (*p* = 0.894). Figures 5 and 6 show examples of physiological patellar tracking and maltracking.

**Figure 7:**
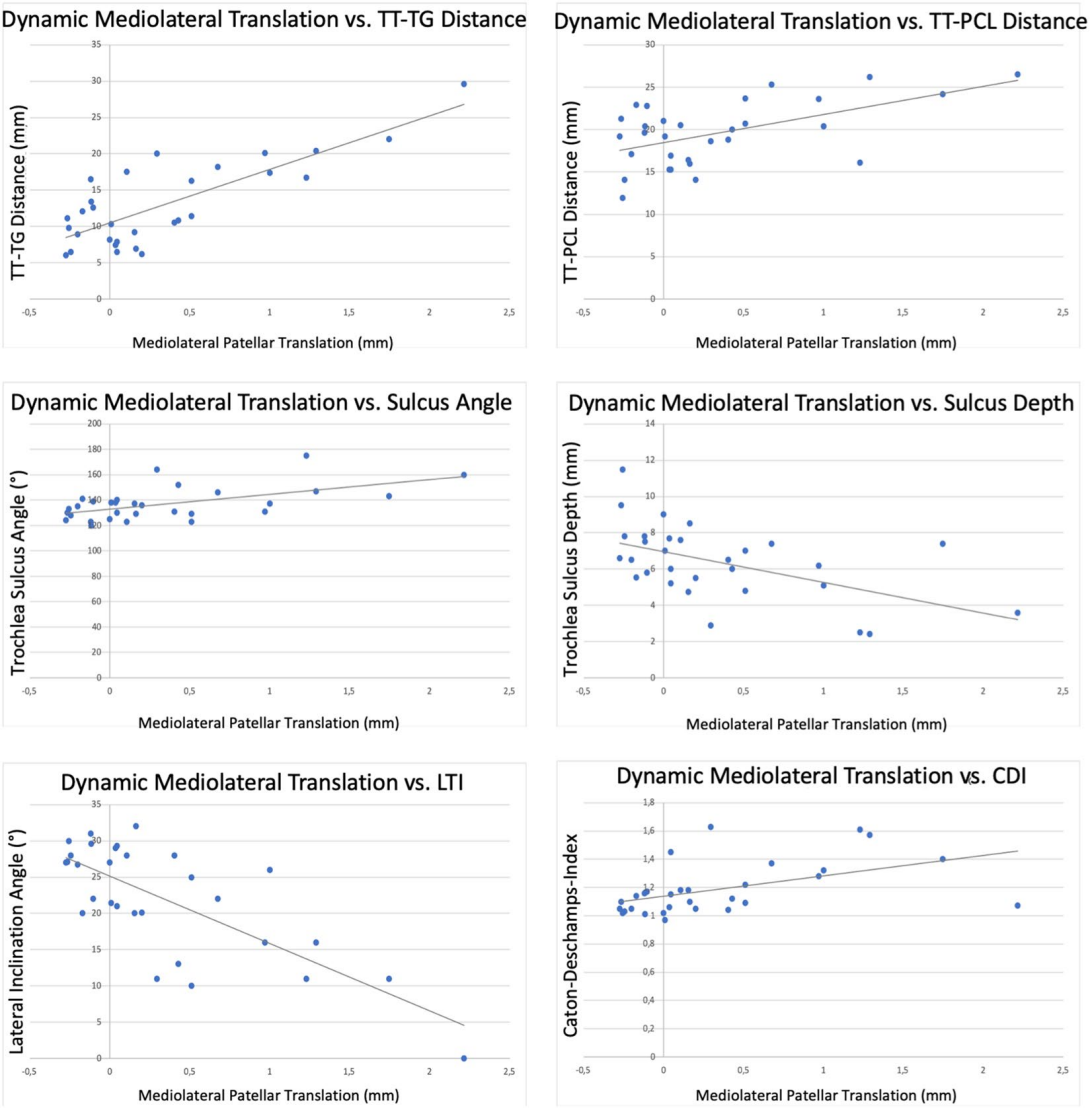
Correlation analysis for common radiological parameters with dynamic mediolateral patellar translation. TT-TG: tibial tuberosity to trochlear groove distance; TT-PCL: tibial tuberosity to posterior cruciate ligament distance; LTI: lateral trochlear inclination angle, CDI: Caton-Deschamps-Index.

The distance of dynamic mediolateral translation correlated strongly with the TT-TG (r = 0.701, *p* < 0.001) and with the lateral inclination angle (r = − 0.728, *p* < 0.001), as well as with the CDI (r = 0.536, *p* = 0.002), the trochlear sulcus depth (r = − 0.567, *p* = 0.001) and the trochlear sulcus angle (r = 0.642, *p* < 0.001). The correlation with the TT-PCL was less strong, but also significant (r = 0.385, *p* = 0.036) (Fig. 7).

**Figure 8:**
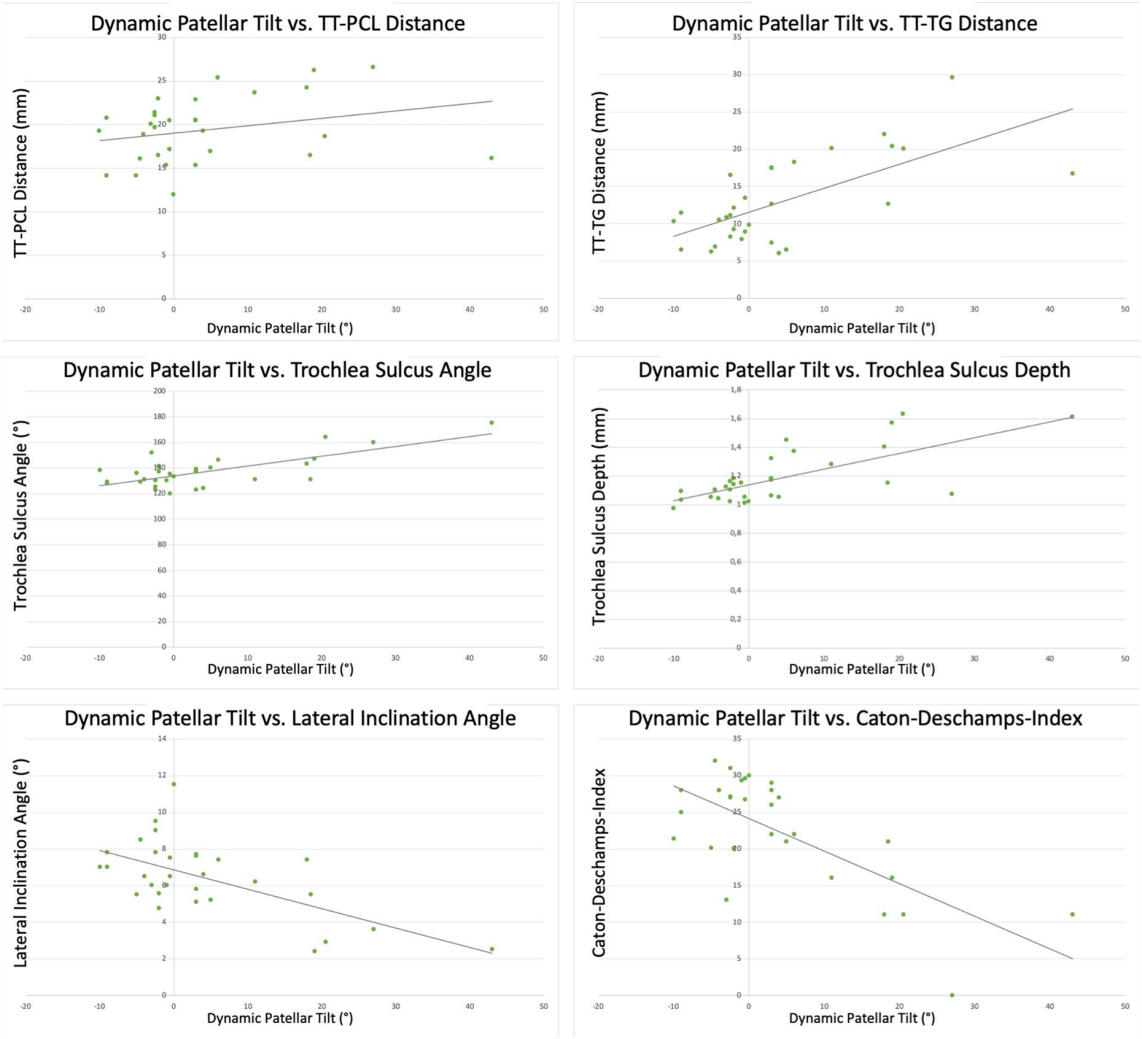
Correlation analysis for common radiological parameters with dynamic patellar tilt. TT-TG: tibial tuberosity to trochlear groove distance; TT-PCL: tibial tuberoisity to posterior cruciate ligament distance; LTI: lateral trochlear inclination angle, CDI: Caton-Deschamps-Index.

The dynamic patellar tilt also correlated with the TT-TG (r = 0.663, *p* < 0.001) and trochlear sulcus depth (r = − 0.620, p < 0.001), and strongly with the CDI (r = 0.716, *p* < 0.001), trochlea sulcus angle (r = 0.725, *p* < 0.001) and the lateral trochlear inclination angle (r = − 0.702, *p* < 0.001) (Fig. 8). Here, no correlation was found with the TT-PCL (r = 0.268, *p* = 0.152).

An excellent interrater correlation was revealed between the two examiners (J.F. and J.M.), regarding dynamic mediolateral translation (ICC 0.971, 95% confidence interval 0.939–0.986) and dynamic patellar tilt (ICC 0.976, 95% confidence interval 0.950–0.988).

The reproducibility of the described measuring technique among the healthy controls was excellent for the dynamic mediolateral translation (2.7 ± 1.6 mm [examination 1] and 3.2 ± 1.2 mm [examination 2]) (ICC 0.923, 95% confidence interval 0.283 – 0.992) and dynamic patellar tilt (6 ± 2° [examination 1] and 8 ± 1° [examination 2]) (ICC 0.921, 95% confidence interval 0.202–0.992)^35^.

## Discussion

The most important finding of this study was that the presented technique allowed fast and reliable measurement of patellar maltracking in symptomatic patients, allowing a clear distinction of a dynamic mediolateral translation and tilt from physiological tracking between patients and healthy individuals. Furthermore, all anatomic risk factors showed strong correlations with both parameters of patellofemoral kinematics.

Generally, the technical possibility of visualizing patellar maltracking with MRI is nothing new and has been demonstrated in previous studies^9,11,15,27^. McNally et al. have pioneered the field of dynamic MRI for patellofemoral diagnostics^27^. They proposed a methodical approach to quantify the amount of patellar lateralization by measuring the distance between the lateral edge of the patella and the lateral rim of the trochlear groove on transversally orientated planes^27^. This technique has been adopted and modified with a simplified examination design, to serve as a radiation-free alternative for dynamic CT examination^21,26^. However, since the technique mentioned above did not consider individual differences of joint size or patella tilt, it is likely to have a certain measurement bias^8,36^. In our presented study, all measurements were referenced to the deepest point of the trochlear sulcus. In addition, the center—center point in the antero-posterior and medio-lateral width was determined and used for measurement, thereby accounting for the patella’s size and tilt.

In a previous cinematic MRI study, Burke et al. found a significantly greater lateralization in patients with patellofemoral instability, when compared to the control group^26^. Although these principle findings were confirmed by the results of the presented study, the measured distance of patellar lateralization was explicitly larger, compared to the results of Burke et al., which may be attributed to differences of anatomical risk factors or the measuring technique^26^. Furthermore, the previous studies failed to provide a sufficient characterization of patellar tracking as such, by leaving the patella position inside the trochlea (knee in flexion) almost unconsidered^26^. However, particularly in this position, no significant differences were noted between asymptomatic and symptomatic subjects. Consequently, the patella was guided securely inside the trochlear groove in both groups. Only with increasing extension, as the patella left the trochlea groove, did symptomatic patients demonstrate a significantly greater dynamic mediolateral translation compared to the control group. This was accompanied by a significantly increased patellar tilt in extension. Based on these findings, proximal patellar maltracking, which takes place proximally to trochlear guidance, was measurable for the first time in actual patients and could be distinguished from physiological patellar tracking^6^.

The emergence of patellofemoral maltracking is frequently associated with the existence of common anatomical risk factors, which determine the indication and planning of surgical procedures^2,9,10,14,17,37^. Most of these risk factors are derived from static MRIs, although their actual influence on patellofemoral kinematics cannot be seen conventionally^25,38–40^.

Burke et al. found a mean TT-TG of 14.5 ± 5.2 mm in their patient collective, which most likely corresponds to a normal value^2,5,6,17,37^. They found only a moderate correlation between TT-TG and the lateral patellar tracking (r = 0.48, *p* = 0.006)^26^; however, both parameters depend on flexion angle and size of the joint^8,36,41^. In the same study, neither the trochlear sulcus depth, nor the patella height showed any correlation at all^26^. In our presented study, the mean values for the Caton-Deschamps Index, TT-TG, and trochlear sulcus depth in the patient group were pathological^2,15,17^. Furthermore, all the measured parameters correlated with the extent of dynamic mediolateral patellar translation and with the dynamic patellar tilt. Specifically, the TT-TG distance, trochlear sulcus angle and lateral trochlear inclination angle showed particularly strong correlation coefficients, while the patella height (CDI) strongly correlated with the dynamic patellar tilt.

These observations support the findings of previous studies, which have identified the trochlear shape and TT-TG as particularly strong predictive parameters for patellofemoral instability^42,43^. In this context, dynamic MRI could provide important valuable additional information to evaluate the influence of present risk factors. Reciprocally, the clear correlations of the above-mentioned parameters with dynamic mediolateral translation and dynamic patella tilt support the validity of the present measuring technique. This was reinforced by an excellent interrater agreement of both kinematic parameters.

In another previous study on dynamic MRI examination, Clark et al. have described a semi-open chain motion setup in order to analyse patellar tracking^29^. The patients were asked to extend their knees against the resistance of a deflating air balloon, while the scans were obtained using a cardiac coil, which was placed on top of the knee. The main objective in the cited study was to examine the congruency in stable and unstable PFJ. Therefore, with respect to this aim, dynamic imaging was focused to the extent of the trochlear groove. The authors found a significant lesser PF contact area in unstable PFJ, respectively^29^. Other authors have used dynamic MRI protocols, to investigate the correlation between patellofemoral morphology and kinematics, as well as the difference to healthy controls. Carlson et al. investigated in the patellofemoral morphology and patellar tracking of female adolescent patients, who showed symptoms of patellofemoral pain^40,44^. However, these measuring techniques did not allow quantification of patellar tracking and the reliability was not tested.

In addition to the improvement of clinical diagnostics, the results of the presented study reveal new aspects of physiological patellar tracking in healthy individuals. In contrast to a popular cadaver study, the maximal physiological patellar lateralization in extension was notably smaller in our control group (3.1 ± 2.4 mm) compared to the value of 5 ± 2.6 mm as described by Amis et al.^1^. Accordingly, the measured distance of dynamic mediolateral patellar translation showed, that the patella of healthy individuals travelled in almost a straight line. This controversy may be explained by an insufficient in-vitro simulation of the medial muscle vector. While the muscle influence is most likely underrepresented in a biomechanical experiment, it has a major influence on patellar tracking in vivo^1,39,45–47^.

In the presented study, permanent muscle activity during image acquisition was ensured by the specific examination setup. The predetermined range of motion was repeated twice, continuously, so muscular relaxation in resting position was effectively excluded. Further, the presented imaging sequence allowed pressure-free and naturally fast knee movement, in order to gain a realistic impression of patellar tracking in the investigated subjects. The utilized imaging sequence comprised Cartesian k-space filling, which allowed the use of parallel imaging, with robust SENSE reconstruction of the data. By contrast, radial k-space filling, as used by Burke et al.^26^ has inherent oversampling of the k-space centre, thereby leading to longer scan times and streaking artefacts, if the difference in position of the knee between acquisition of neighbouring spokes in k-space is too large. Consequently, radial imaging requires a slower course of knee movement.

In the study by Burke et al.^26^, this resulted in a scan time period of 2 min for a motion between 30° of flexion and full extension. During this time, multiple individual acquisitions were performed. They were later repositioned, in order to cover the full range of patellofemoral tracking. Further, all dynamic acquisitions were performed with an abdomen coil, which was placed on top of the knee movement, in possible interference with the moving patella^26^. Therefore, the resulting patellofemoral articulation is artificial and presumably inconsistent with reality.

With the use of cartesian k-space filling, the dynamic sequence was reduced to a time frame of 30 s. During this time, the same range of motion was performed twice at a steady and realistic pace. This entire motion sequence was acquired with three simultaneous transverse slices, which dispensed with retrospective slice repositioning and resulted in one complete real-time motion sequence. The use of two ring coils, which were positioned at both sides of the knee, eliminated the possibility of mechanical interference with the patella. Moreover, a higher in-plane resolution (1.6 × 1.6 mm^2^ compared to 2.0 × 2.0 mm^2^) and slightly thinner slices (7 mm versus 8 mm) could be achieved, resulting in a higher image quality. Compared to a standard static MRI imaging protocol, which took 13 min 17 s in this case, additional cinematic sequences extended the examination time only by 95 s, so a total duration of 15 min was not exceeded.

The findings of the presented study demonstrate, that significant differences of PF articulation can be visualized between healthy individuals and symptomatic patients. In the future, objectification of patellofemoral tracking may be particularly helpful in cases of clinical doubt and borderline indications of surgical techniques. It may also serve as a tool for complication analysis, in case of persisting symptoms postoperatively. Furthermore, the integration of natural muscle activity considerably enhances its clinical significance and contributes to a more realistic understanding of the dynamic interaction between different anatomical factors. Compared to clinical examination, the results of this measuring technique further show a very high interrater-reliability and reproducibility, which might improve patellofemoral diagnostics in general. At the same time, the technical requirements are manageable, while the required acquisition time is short. This makes this technique a convenient addition to conventional MRI examination.

The definition of normal values should be the goal of future studies, to support surgical indications or to perform a postoperative control of success in the future.

## Limitations

The present study had some limitations.

One was that the dynamic MRI imaging of the patellofemoral joint is not yet considered a generally available diagnostic tool and therefore does not provide gold standard examination protocols or measuring techniques. Compared to CT-based protocols, MRI is limited by acquisition time and slice thickness, which requires a thoughtful choice of the applied slice orientation to capture all relevant landmarks. Accordingly, measurements may be biased by unintentional movement of the knee inside the coil, which was addressed by the use of a customized positioning device. With future technical advances, a time-efficient use of three-dimensional blocks, similar to CT scans, will be beneficial. Another limitation is that the possible range of motion was limited by the diameter of the gantry, allowing a maximum flexion angle of 40°–45°. For the purpose of characterizing a proximal patellar maltracking, however, a range of motion between 0° and 40° appeared to be sufficient, as this range covers the patellotrochlear engagement. Furthermore, TT-TG measurement can be biased by variable joint sizes, which can be addressed with the use of indices. However, until today these have not yet been clinically validated. No power analysis was performed prior to the study, given the fact that there were no reference values available for this novel measuring technique. The sample size of both groups was relatively small and may therefore present further bias. The patient group showed imbalances in favour of female patients. However, this reflects the general gender distribution for patellar dislocations. A follow-up study with larger subgroups will also be necessary to allow a distinct differentiation between the individual influences of the presented risk factors.

## Conclusion

The present study demonstrates that dynamic MRI is a reliable way to differentiate patellofemoral maltracking from physiological patellar tracking in healthy individuals. Technical advances will allow a more complete observation and measurement of realistic patellofemoral kinematics, under natural biomechanical conditions and physiological muscle activity.

The TT-TG distance, trochlea sulcus depth and patella height are particularly important predictive parameters and strongly correlate with dynamic mediolateral patellar translation and patellar tilt. Therefore, a thorough analysis of present anatomical risk factors for patellar maltracking is favourable for clinical decision making.
